# Cortico-striatal beta oscillations as a reward-related signal

**DOI:** 10.3758/s13415-024-01208-6

**Published:** 2024-08-15

**Authors:** M. F. Koloski, S. Hulyalkar, S. A. Barnes, J. Mishra, D. S. Ramanathan

**Affiliations:** 1 Mental Health Service, VA San Diego Healthcare Syst, La Jolla, CA USA; 2 Department of Psychiatry, UC San Diego, La Jolla, CA USA

**Keywords:** Beta oscillations, Cortico-striatal network, Delay discounting, Electrical stimulation, Local field potentials

## Abstract

The value associated with reward is sensitive to external factors, such as the time between the choice and reward delivery as classically manipulated in temporal discounting tasks. Subjective preference for two reward options is dependent on objective variables of reward magnitude and reward delay. Single neuron correlates of reward value have been observed in regions, including ventral striatum, orbital, and medial prefrontal cortex. Brain imaging studies show cortico-striatal-limbic network activity related to subjective preferences. To explore how oscillatory dynamics represent reward processing across brain regions, we measured local field potentials of rats performing a temporal discounting task. Our goal was to use a data-driven approach to identify an electrophysiological marker that correlates with reward preference. We found that reward-locked oscillations at beta frequencies signaled the magnitude of reward and decayed with longer temporal delays. Electrodes in orbitofrontal/medial prefrontal cortex, anterior insula, ventral striatum, and amygdala individually increased power and were functionally connected at beta frequencies during reward outcome. Beta power during reward outcome correlated with subjective value as defined by a computational model fit to the discounting behavior. These data suggest that cortico-striatal beta oscillations are a reward signal correlated, which may represent subjective value and hold potential to serve as a biomarker and potential therapeutic target.

**Supplementary information:**

The online version contains supplementary material available at 10.3758/s13415-024-01208-6.

## Introduction

Reward processing comprises the set of neural systems that encode appetitive, motivational, or pleasurable stimuli (Berridge & Kringelbach, 2008; Dalley et al., 2004). Information about reward outcomes is used to inform future decisions, and deficits in reward processes are linked with learning and decision-making impairments and likely contribute to anhedonia, amotivation, and substance abuse problems observed in various psychiatric conditions (Dalley et al., 2004; Pujara & Koenigs, 2014). The value associated with a positive outcome is sensitive to external factors, such as the time between the choice and reward delivery. This concept can be evaluated in humans and animals by using temporal discounting tasks to measure preference for a small reward delivered immediately or a larger reward delivered after a delay (Le Van Quyen et al., 2001). Reward is devalued by increasing temporal delays, but the rate at which reward is devalued may be different for everyone (Winstanley et al., 2004; Kable & Glimcher, 2007; Kobayashi & Schultz, 2008; Roesch et al., 2011; Lefner et al., 2021; Story et al., 2016). Current clinical diagnostics and treatments are not well suited for these individual differences in symptoms and pathology. Discovering a reliable biomarker that signals reward processing deficits would provide information about specific brain regions or brain states to target during treatment (Farzan, 2023).

In rodents, the medial prefrontal cortex exerts top-down control over reward-related areas, such as orbitofrontal cortex, ventral striatum, and basolateral amygdala through cortico-striatal-limbic projections (Abler et al., 2006; Bayer & Glimcher, 2005; Berridge & Kringelbach, 2008; Dalley et al., 2004; Groenewegen et al., 1997; Haber & Knutson, 2010; Salehinejad et al., 2021; Schoenbaum et al., 2009; Schultz et al., 2000). This extended “reward” network is innervated by midbrain dopamine neurons originating from the ventral tegmental area, which contribute to reward processing behaviors through reward-prediction error signals (the difference between expected and actual rewards) (Bayer & Glimcher, 2005; Berridge & Robinson, 1998; Francois et al., 2014; Haber & Knutson, 2010; Humphries & Prescott, 2009; Kobayashi & Schultz, 2008; Snyder et al., 2020). Several lines of research suggest that cortico-striatal circuitry is important for reward processing and activated during temporal discounting. Single neurons in prefrontal/orbitofrontal cortex and ventral striatum modulate activity during reward anticipation and delivery (Atallah et al., 2014; Constantinople et al., 2019; Francoeur & Mair, 2018, 2019; Goldstein et al., 2012; Levcik et al., 2017; van der Meer & Redish, 2009; van Duuren et al., 2009) and can be modulated by different types (Carelli et al., 2000; Schultz et al., 2000), magnitudes (Goldstein et al., 2012; Roesch et al., 2011; Schultz et al., 2000; Simon et al., 2015), and locations of reward (Francoeur & Mair, 2018; van der Meer & Redish, 2009). Neurons from any brain region can encode a diverse array of task-related processes and, alone, may not accurately reflect the larger scale network-wide activity that occurs during reward processing (Cui et al., 2016; Francoeur & Mair, 2018; MacDowell & Buschman, 2020; Williams et al., 2018). In humans, activity in the cortico-striatal network (LOFC and ventral striatum) is involved in reward processing (Ballard & Knutson, 2009; Boettiger et al., 2007). Specifically, during temporal discounting, oscillatory activity at theta and beta frequencies is associated with a preference for larger, delayed rewards (Pornpattananangkul & Nusslock, 2016). Changes in oscillatory activity at distinct frequencies can indicate behavioral and disease states, and predict an individual’s response to treatment (Buzsáki & Watson, 2012; Masimore et al., 2004). Growing evidence supports a role for theta oscillations in cognitive-control processes and beta for reward feedback (Cohen et al., 2007; HajiHosseini & Holroyd, 2015; Marco-Pallares et al., 2008; Marco-Pallarés et al., 2015; Patai et al., 2022; Pornpattananangkul & Nusslock, 2016; Zavala et al., 2018), both necessary for successful performance on a temporal discounting task.

Local field potentials (LFP) offer an opportunity to bridge micro- and macroscopic levels of brain activity across the reward network and may provide a more robust, stable, and simpler framework to identify neurobehavioral relationships that can be compared with human neuroimaging and electrophysiological investigations (Williams et al., 2018; MacDowell & Buschman, 2020; Abbaspourazad et al., 2021; Cacioppo et al., 2008). Finding a physiological biomarker linked with reward processing can help to uncover the basic mechanisms of value-based decision-making and may offer a potential therapeutic target for disorders where decision-making is impaired. We have previously used multisite LFP recordings to characterize networks operating at distinct oscillatory frequencies to support behavioral inhibition and default-mode-like processing (Fakhraei et al., 2021a, 2021b). We utilized our multisite LFP approach to identify electrophysiology markers linked with delay discounting behavior during reward outcome. We chose the reward outcome period (and not trial onset, response, or delay period) to specifically examine reward valuation processes opposed to choice, anticipation, or time estimation signals (Kable & Glimcher, 2009). In temporal discounting, value is subjectively attributed to an outcome based on objective measures of reward magnitude and temporal delay (Kable & Glimcher, 2007). Beta activity in cortico-striatal-limbic electrodes sites was sensitive to reward magnitude and temporal delay and correlated with subjective value as defined by a computational model. Lastly, we provide preliminary evidence that modulating beta activity with “on-demand” electrical stimulation can influence choice behavior. Our evidence suggests that beta oscillations may be a translationally relevant signal of reward value that can help to identify individual differences in reward processing and offer a new therapeutic target (Berridge & Kringelbach, 2008; Bilderbeck et al., 2020; Pujara & Koenigs, 2014; Whitton et al., 2015).

## Materials and methods

### Ethics statement

This research was conducted in strict accordance with the Guide for the Care and Use of Laboratory Animals of the National Institutes of Health. The protocol was approved by the San Diego VA Medical Center Institutional Animal Care and Use Committee (IACUC, Protocol Number A17-014).

### Experimental design

#### Subjects

Eighteen Long-Evans rats (15 males) obtained from Charles River Laboratories were used for these experiments. When received, rats were ~ 1 month and weighed 150 g. Habituation and pretraining was initiated 2 weeks after arrival. Rats were housed in pairs before electrode implantation and individually housed thereafter in a standard rat cage (10 × 10.75 × 19.5 in, Allentown, NJ) with free access to food and on a standard light cycle (lights on at 6 am/off at 6 pm). During behavioral training, animals underwent water scheduling (free access to water for 2 h/day) to maintain motivation for water reward in the tasks. Water was unrestricted on nontraining days and rats were weighed weekly to ensure that water scheduling did not lead to reduced food intake. Subjects with chronic implants were monitored daily for signs of infections, injuries, and bleeding.

#### **Operant chamber and training**

We used a custom-designed operant chamber equipped with five nose-ports (NP), each with an LED, IR sensor, and metal cannula for water delivery. The chamber also contained two auditory tone generators, a house-light, a screen to display visual stimuli, and five peristaltic stepper motors/water pumps that delivered the water rewards into NPs. The chamber was 16 × 12 × 16 (L x W x H) inches with a ceiling opening that allowed electrophysiology tethers to move freely. Simulink (Mathworks) installed directly onto a Raspberry Pi system controlled the behavioral programs. Behavioral outputs from the operant chambers were synchronized with electrophysiological signals using lab-streaming-layer, a protocol designed to integrate multiple behavioral and physiological streams into a common timing stream (Buscher et al., 2020; Ojeda et al., 2014). The design, operation and software control of this chamber has been described previously (Buscher et al., 2020). Animals first went through a pretraining period (5–10 sessions), to learn that a NP with an LED “on” signaled an available response port. That responding in an available NP would trigger a water reward and finally that there was a sequential nature to the task (animals start a “trial” by first entering the middle NP (3), after which they could use either of the neighboring ports (2 or 4) to respond and collect an immediate reward). Animals advanced to the next stage of training when they consistently performed ≥ 100 trials in a 60-min session.

#### Temporal discounting task

Generally, temporal discounting tasks center around choosing between a low-value reward delivered immediately or a high-value reward delivered after a delay. In our version of the task, subjects chose between a small (1x) reward delivered after a fixed short delay (500 ms after response) or a large (3x) reward delivered after a fixed delay that varied from session to session. Each session began with 6 forced-choice trials, orienting the rat to both the low-value (NP 2) and high-value (NP 4) options. During these forced choice trials, the houselight was on and LED lights signaled the available response port, alternating between response port 2 (low-value) and 4 (high-value). A reward was delivered after each response with a fixed delay of 500 ms. Rewards were always delivered from NP 3 and consisted of either 10 µl of water (delivered at the rate of 10 µl/s) following the low-value response selection or 30 µl of water (delivered at the rate of 30 µl/s) following the high value selection. The forced choices helped remind animals each day of small and large reward magnitudes that would be delivered following a selection of NP 2 versus NP 4. After the 6 trials were complete, the houselights dimmed and rats began the full, self-paced, trial sequence. Each trial began with LEDS from response ports (NP 2 and 4) on. Response in the low-value response port (NP 2) turned on the houselights, the middle NP LED (3), and a tone (500-ms duration) to indicate a choice was made. A small reward (10 µl delivered over a 1-s duration) was delivered 500 ms after the response (immediately after the tone ended) from NP 3. Selecting the high-value response port (NP 4) turned on the houselights, the middle NP LED (3), and a tone (500-ms duration). The tone and NP LED occur 500 ms before reward delivery. The motor makes an audible sound during water delivery and is the only cue paired with reward, and that signals the duration of reward (1 s vs. 3 s). Each session had a different delay following the high-value selection, and that delay was held constant for the entire session. Possible delays the animal could experience following selection of the high-value port included: (0.5 s, 1 s, 2 s, 5 s, 10 s, 20 s). Following the delay selected for that session, a large reward (30 µl over a 3-s duration) was delivered from NP 3. The high-value delay alternated between behavioral sessions but remained the same throughout the entire (60 min) session. The houselights turned off when water was delivered out of NP3 and a 5-s intertrial interval began after water delivery.

Training was performed prior to testing on this task. First, for 3 days the animals were acclimatized to the operant chamber and trained to associate responses in any of the 5 NPs with rewards (at this stage all responses led to the same 20-µl reward). In the next phase rats learned to discriminate choices based on reward magnitude. The task structure described above was used, but an identical delay post response (500 ms) was used for both the high (NP4) and low-value (NP2) responses. Once animals expressed a clear preference (≥ 70%) for the large reward and consistently performed ≥ 100 trials, they were advanced to train on the other delay conditions. Animals that did not show a clear preference for the large reward after 15 training sessions (3 weeks) were excluded from the study. Animals who advanced through training then underwent surgical implantation of electrodes as described below, followed by data collection on the task. Training on average lasted 18 sessions across 5 weeks. After surgical electrode implantation we waited two weeks to allow animals to recover from surgery before electrophysiology recording began.

#### Electrophysiology recording

Twelve male Long-Evans rats were used to collect large-scale local field potential (LFP) data during the temporal discounting task. LFP data was recorded using a 32-channel RHD headstage (Intan Technologies, CA, USA; Part C3324) coupled to a RHD USB interface board (Intantech, Part C3100) and SPI interface cable. We used plug-in GUI (Open Ephys) software for acquisition. Data was recorded at 1 kHz, with a band-pass filter set at 0.3 to 999 Hz during acquisition. Physiology data were integrated with behavioral data by using a lab-streaming-layer (LSL) protocol (Ojeda et al., 2014), as described previously (Buscher et al., 2020). Our analyses focused on 12 electrodes (Table 1). Recording sessions lasted 60 min and occurred 3–4 days per week. Analyses are based on data from 148 behavioral sessions (124 with electrophysiology) across 12 rats. There was an average of two sessions/delay/subject (n = 23 sessions at 0.5 s; n = 26 sessions at 1 s; n = 17 sessions at 2 s; n = 28 sessions at 5 s; n = 33 sessions at 10 s; n = 21 sessions at 20 s). Rats were 8 months at the end of recording.

**Table 1:** List of 12 electrode sites

Abbreviation	Brain area
M2	Secondary motor cortex
A32D	Dorsomedial prefrontal cortex
A32V	Ventromedial prefrontal cortex
vOFC	Ventral orbitofrontal cortex
ALM	Anterolateral motor cortex
LFC	Lateral frontal cortex
Ains	Anterior insula
lOFC	Lateral orbitofrontal cortex
VMS	Ventromedial striatum
NAcS	Nucleus accumbens shell
NAcC	Nucleus accumbens core
BLA	Basolateral amygdala

#### **Electrical stimulation**

In six rats (3 males), we tested “on-demand” electrical stimulation applied based on the rodent’s choice behavior on the temporal discounting task (30-min sessions). We applied electrical stimulation during the reward outcome period of large reward choices (3 s). Beta frequency stimulation (20 Hz) was applied to one of the 12 cortico-striatal electrode sites chosen based on electrode impedance (target 30–90 kOhm). Stimulation was applied with the Tucker-Davis Technologies (TDT, FL, USA) IZ2H system. The 32-CH omnetics EIB board (described in methods) was fitted with an adapter (ZCA-OMN32) to connect a 16-Ch ZIF-clip. The ZIF-clip connected to the stimulator through a motorized commutator (AC032, TDT) with an SPI interface cable. Synapse (TDT) software controlled the stimulation parameters and integrated stimulation with behavioral markers through Simulink (MATLAB). Stimulation started with reward onset and lasted for the duration of reward (3 s) and consisted of a biphasic 20-Hz pulse with 40-ms duration between each bipolar pulse. Current changed based on impedance for each individual rat (35–80 µA). Likewise, target electrode changed based on sites available within target impedance range (see Table S3 for individual parameters). Rats had undergone training on delay discounting for another study (data not reported) and were approximately 9 months at the start of the stimulation study.

The task was modified to include one, fixed large reward delay to ensure the rat completed enough trials to see a statistically significant effect of stimulation. The temporal delay chosen for stimulation was adjusted for each subject to ensure that, without stimulation, selection of high-value choice occurred on average at least 30% of the time (and no more than 70%) to have some degree of stimulation built into the paradigm (i.e., 1-s and 20-s delays create ceiling effects where rats are making too few small or large choices). The temporal delay for the high-value reward was thus different for each subject (2 s, 5 s, or 10 s) but remained consistent throughout all stimulation sessions for each animal. The delay for the small reward was fixed at 0.5 s. Animals included for stimulation were well-trained (3 months) on the temporal discounting task and had experienced at least 2 sessions at each delay condition before beginning this experiment. During this experiment, each subject had three consecutive baseline sessions (no stimulation), three stimulation sessions, and three recovery sessions (no stimulation).

### Surgery

Aseptic surgeries were performed under isoflurane anesthesia (SomnoSuite, Kent Scientific, CT) with all instruments autoclaved before start. Animals received a single dose of atropine (0.05 mg/kg) to diminish respiratory secretions during surgery, a single dose of dexamethasone (0.5 mg/kg) to decrease inflammation, and 0.5–1 ml of 0.9% sodium chloride solution before surgery. The area of incision was cleaned with 70% ethanol and iodine solution. A local anesthetic, Lidocaine (max. 2 cc), was injected under the skin at the incision site while the animal was anesthetized but before surgery initiation.

The fabrication and implantation procedures of our custom fixed field potential and single-unit probes are described in detail (Francoeur et al., 2021). Briefly, our LFP probe targets 32 different brain areas simultaneously; 50-µm tungsten wire (California Fine Wire, CA, USA) used for our electrodes was housed in 30-gauge stainless steel metal cannula (Mcmaster-Carr, Elmhurst, IL) cut 8-9 mm long. Each cannula (N = 8) contained four electrode wires cut to their unique D/V length (Fig. S1). The average impedance of our blunt-cut tungsten microwires is 50 kOhms at 1 kHz. During surgery, eight holes were drilled in the skull (1 for each cannula) at predetermined stereotactic locations. Additional holes were drilled for a ground wire and anchor screws (3–8). The ground wire was soldered to an anchor screw and inserted above cerebellum. Electrodes were slowly lowered to desired depth, pinned to the EIB board, and secured with superglue followed by Metabond (Parkell, NY). The entire head stage apparatus was held to the skull and encased with dental cement (Stoelting, IL).

At the conclusion of surgery, the skin was sutured closed, and rats were given a single dose (1 mg/kg) of buprenorphine SR for pain management. Rats recovered from surgery on a heating pad to control body temperature and received sulfamethoxazole and trimethoprim in their drinking water (60 mg/kg per day for 8 days) to prevent infections.

Rats in the stimulation experiment underwent identical surgical procedures. At the end of their LFP recording experiment (data not included here), they were put under isoflurane anesthesia for ~ 5 min to add the external adapter (ZCA-OMN32) to connect a 16-Ch ZIF-clip. Dental cement was carefully placed to secure the connection between the adapter and EIB board. Rats were observed until they were ambulatory and eating/ drinking.

### Statistical analysis

#### **LFP Time Frequency Analysis**

We performed standard pre-processing and time–frequency (TF) analyses using custom MATLAB scripts and functions from EEGLAB (Fakhraei et al., 2021a, 2021b; Francoeur et al., 2021).

1. Data epoching: We first extracted time-points for events of interest. This paper focused on neural activity time-locked to reward (opposed to trial onset, choice, or delay period). Because of the number of comparisons (frequencies, electrodes), we had to focus on one specific time period. Our motivation was to examine neural signals during reward outcome as is common in neurophysiological studies of reward processing during probabilistic or delayed reward conditions (HajiHosseini & Holroyd, 2015; Iturra-Mena et al., 2023; Marco-Pallarés et al., 2015; Pornpattananangkul & Nusslock, 2016; Walsh & Anderson, 2012). Time-series data were extracted for each electrode (32), for each trial and organized into a 3D matrix (electrodes, times, trials).
2. Artifact removal: Activity was averaged across the time/electrodes to get a single value for each trial. Trials with activity greater than 4X standard deviation were treated as artifact and discarded.
3. Median reference: At each time-point, the “median” activity was calculated across all electrodes (32) and subtracted from each electrode as a reference.
4. Time–Frequency Decomposition: A trial by trial time–frequency decomposition (TF decomposition) was calculated using a complex wavelet function implemented within EEGLAB (newtimef function, using Morlet wavelets, with cycles parameter set to capture frequency windows of 2 to 150 Hz and otherwise default settings used. We calculated the analytic amplitude of the signal (using the abs function in MATLAB).
5. Baseline normalization: To measure evoked activity (i.e., change from baseline) we subtracted, for each electrode at each frequency, the mean activity within a baseline window (1000–750 ms before the start of the trial).
6. Trial averaging: We next calculated the average activity across trials for specific trial types at each time-point and frequency for each electrode, thus creating a 3D matrix (time, frequency, and electrode) for each behavioral session. We separated trials based on choice (high vs. low-value port) and delay condition.
7. Comparison across animals: Before averaging across sessions/animals, we “z-scored” the data recorded from each behavioral session by subtracting the mean and dividing by the standard deviation of activity in each electrode (at each frequency) over time. Z-scoring was helpful for normalizing activity measured from different animals prior to statistical analysis. Because we had already performed a “baseline” subtraction (as described above), this analysis captured whether there was a significant increase or decrease in activity compared to baseline. FDR-correction was performed across all time–frequency-electrodes (FDR-corrected *p*-value threshold set to 0.05). These pre-processing steps resulted, for each session, in a 3D time–frequency-electrode matrix of dimensions 200 × 139 × 32, which was used for further statistical analyses as described below.

#### LFP Weighted Phase-Lagged Index (wPLI) Analysis

wPLI is a method developed to be less sensitive to noise, volume conduction, and other artifacts compared with standard methods (such as coherence) (Vinck et al., 2011). Phase-coherence or phase synchrony has long been thought as a method to measure some relationship between two signals for brain analyses (Le Van Quyen et al., 2001). However, phase-coherence can be artificially induced by noise, common sources, and volume conduction. For these reasons, novel methods have been developed to minimize these spurious contributors, including imaginary coherence (Nolte et al., 2004) and phase-lagged index (PLI) (Stam et al., 2007). Imaginary coherence uses the imaginary part of the coherence, which is 0 when spurious sources are likely to have the largest contributor to coherence (at 0 and 180 degree angle phase-lags) and is largest when there is a 90° phase-lag). However, this method is less sensitive to smaller but highly consistent phase-lagged relationships. phase-lagged index is a measure of the asymmetry that occurs (lagging vs. leading) between two signals (Stam et al., 2007) and is thus more sensitive to consistent relationships independent of magnitude. The wPLI weights the PLI by the magnitude of the imaginary coherence, thus providing some of the advantages of each of these methods (Vinck et al., 2011). Work since then has suggested that wPLI is at least as good as other methods (in terms of sensitivity to detect changes) while being more robust to noise/spurious correlations induced by volume conduction or common sources (Imperatori et al., 2019; Lau et al., 2012). Data was pre-processed identically to that noted above (epoching, artifact removal and median referencing). We next took the epoch from 0-1 s post-reward delivery and estimated the wPLI across this epoch of time. Finally, we calculated the mean wPLI for each pair-wise interaction at beta-frequencies within each session. Thus, for each session we computed a 12 × 12 symmetric matrix of the wPLI, separately for the high-value rewarded trials and the low-value rewarded trials.

#### **Computational Modeling**

To investigate mechanisms that drive behavior, we estimated how the subjective value attributed to the large reward varied as a function of the delay associated with obtaining it. The value assigned to options is influenced by objective properties (magnitude and delay) but also by internal factors (motivation) (Lak et al., 2014; Schultz, 2015). We initialized the subjective value for each reward magnitude to 30 and 10 (reflecting the absolute volume of each reward size). We fitted three separate models to the behavioral data that utilized a linear, exponential, or hyperbolic discount function to adjust the subjective value of the large reward depending on the delay period.

$$\begin{array}{c}\text{Linear}: SV\left(t\right)=R(t)\bullet (1-k\bullet D\left(t\right))\\ \text{Hyperbolic}: SV\left(t\right)=R(t)\bullet \frac{1}{1+k\bullet D(t)}\\ \text{Exponential}: SV\left(t\right)=R(t)\bullet {e}^{-k\bullet D(t)}\end{array}$$

For each model, the subjective value (SV) of the action selected on trial *t*. *R* denotes the reward magnitude obtained (10 or 30 µl) and *D* denotes the delay period associated with each reward (0, 1, 2, 5, 10, or 20 s for the large reward; 0 s for the small reward). The discount parameter, *k*, controls the steepness of the discount rate. Specifically, an increased discount rate results in a greater reduction in the subjective value attributed to the large reward as the delay period increases.

For each method, we estimated the discount rate for individual subjects. Once the subjective value for each action had been estimated, we used a softmax equation to convert action value into choice probabilities. The degree to which choices are exploratory (i.e., selecting the lower-valued action) vs. exploitative (i.e., selecting the higher-valued action) is controlled by the inverse temperature (β) parameter. A higher β parameter indicates a greater tendency to engage in exploitative choices whereas a lower β parameter is indicative of a greater tendency to explore actions associated with a lower value. Thus, the probability of choosing option A over option B, given the subjective value attributed to each option, can be determined according to:

$$p\left(A\right)= \frac{{e}^{SVA \times \beta }}{{e}^{SVA \times \beta }+ {e}^{SVB \times \beta }}$$

The optimal model parameter values for each of the three models were identified by minimizing the negative log-likelihood of choice probabilities using the “minimize” optimization function with the truncated Newton algorithm in Python’s Scipy library (vs. 1.5). To sample broadly from the range of possible parameter values and reduce the possibility of the model getting stuck in local minima, the optimization algorithm was initialized at 20 different starting points within the parameter space for each subject. The parameter values associated with the lowest negative log-likelihood were selected as the best-fitting set for each subject. The best-fitting model was determined by comparing the corrected Akaike Information Criteria (AIC) value for each model. To confirm the validity of the best-fitting model we conducted posterior predictive checks and compared the simulated performance with actual rodent performance. Moreover, parameter recovery exercises were also performed to confirm the accuracy of parameter estimation.

#### Statistical Tests

Behavior on the temporal discounting task was analyzed in IBM SPSS Statistics version 28 (NY, USA) as a two-way ANOVA. Percent high value choice (dependent variable) was measured for each delay condition (0.5, 1, 2, 5, 10, 20 s), subject, and the delay*subject interaction. A *p* value < 0.05 was considered significant.

We analyzed the time–frequency-electrode data at the level of each session using linear mixed models (LMM) in SPSS to account for subject and session variance and missing data points. To account for the variable delay-to-reward conditions in the temporal discounting task, data were time-locked to reward delivery. We analyzed the first second of activity post-reward (0–1000 ms after reward onset) to control for the difference in water delivery between the large (3 s) and small (1 s) reward magnitude durations. First, we generated a LMM to compare normalized power (dependent variable) of the lOFC electrode at each oscillatory frequency band and trial type (high or low value choice). We used the following frequency bands: delta power (1–4 Hz), theta (4–8 Hz), alpha (8–12 Hz), beta (15–30 Hz), gamma (40–70 Hz) and high gamma (70–150 Hz). [Fixed: Frequency, Trial Type. Random: (Frequency |Subject), Time] with frequency and trial type analyzed as repeated measures. Frequency band was assigned to the slope parameter of the random effect for each subject to account for the interdependence of power at each frequency band. Based on this first test, we fit a second LMM to explore power (dependent variable) at frequencies of interest across 12 electrodes (M2, A32D, A32V, vOFC, ALM, LFC, Ains, lOFC, VMS, NAcS, NAcC, BLA) and trial type (high or low value choice). [Fixed: Delay (0.5, 1, 2, 5, 10, 20 s), Trial Type (High or Low-Value Choice), Electrode (M2, A32D, A32V, vOFC, ALM, LFC, Ains, LOFC, VMS, NAcS, NAcC, and BLA). Delay*Trial Type*Electrode Interaction. Random: Subject, Time]. To determine the best fit of each model, we measured the Akaike information criteria (AIC) and Bayesian information criterion (BIC) of four commonly used covariance models: compound symmetry, scaled identity, AR(1), and unstructured (Magezi, 2015; Maxwell & Delaney, 2004). The scaled identity model, assuming repeated measures may be independent but with equal variance (Magezi, 2015; Maxwell & Delaney, 2004), provided the lowest AIC and BIC scores. We used a restricted maximum likelihood (REML) model with the Satterthwaite approximation in SPSS. Significant interactions were followed up with pairwise comparisons (Bonferroni corrected) in SPSS.

To test the effects of stimulation, our main outcome variables were proportion of high reward choices and number of trials. We used a repeated measures two-way ANOVA to test the within-subject effects of condition (baseline, stimulation, recovery) and session (3 sessions of each condition). Significant main effects or interactions were followed with paired samples *t*-tests, Bonferroni corrected. A *p* value < 0.05 was considered significant. Statistics were run in SPSS, and data were visualized with GraphPad Prism.

### Histology

At completion of recording sessions, wire tips were marked by passing 12 $\upmu$ A current for 10 s through each electrode (Nano-Z, Neuralynx, MO). Rats were sacrificed under deep anesthesia (100 mg/kg ketamine, 10 mg/kg xylazine IP) by transcardiac perfusion of physiological saline followed by 4% formalin. Brains were extracted and immersed in 4% formalin for 24 h and then stored in 30% sucrose until ready to be sectioned. Tissue was blocked in the flat skull position using a brain matrix (RWD Life Science Inc., CA, USA). Brains were sectioned frozen in the coronal plane at 50 $\upmu$ m thick. Brain slices were Nissl stained using thionine to identify the course of the electrode tracks. Sections were processed with a slide scanner at 40 × magnification (Zeiss, Oberkochenn, Germany; Leica Biosystems, IL, USA). Positions of electrodes were inferred by matching landmarks in sections to the rat atlas (Paxinos & Watson, 2013) when electrode tips could not be identified (Fig. S1).

## Results

### *Beta* Power Reflects Reward Processing

#### **Large Reward Preference Decreased at Longer Temporal Delays**

**Fig. 1:**
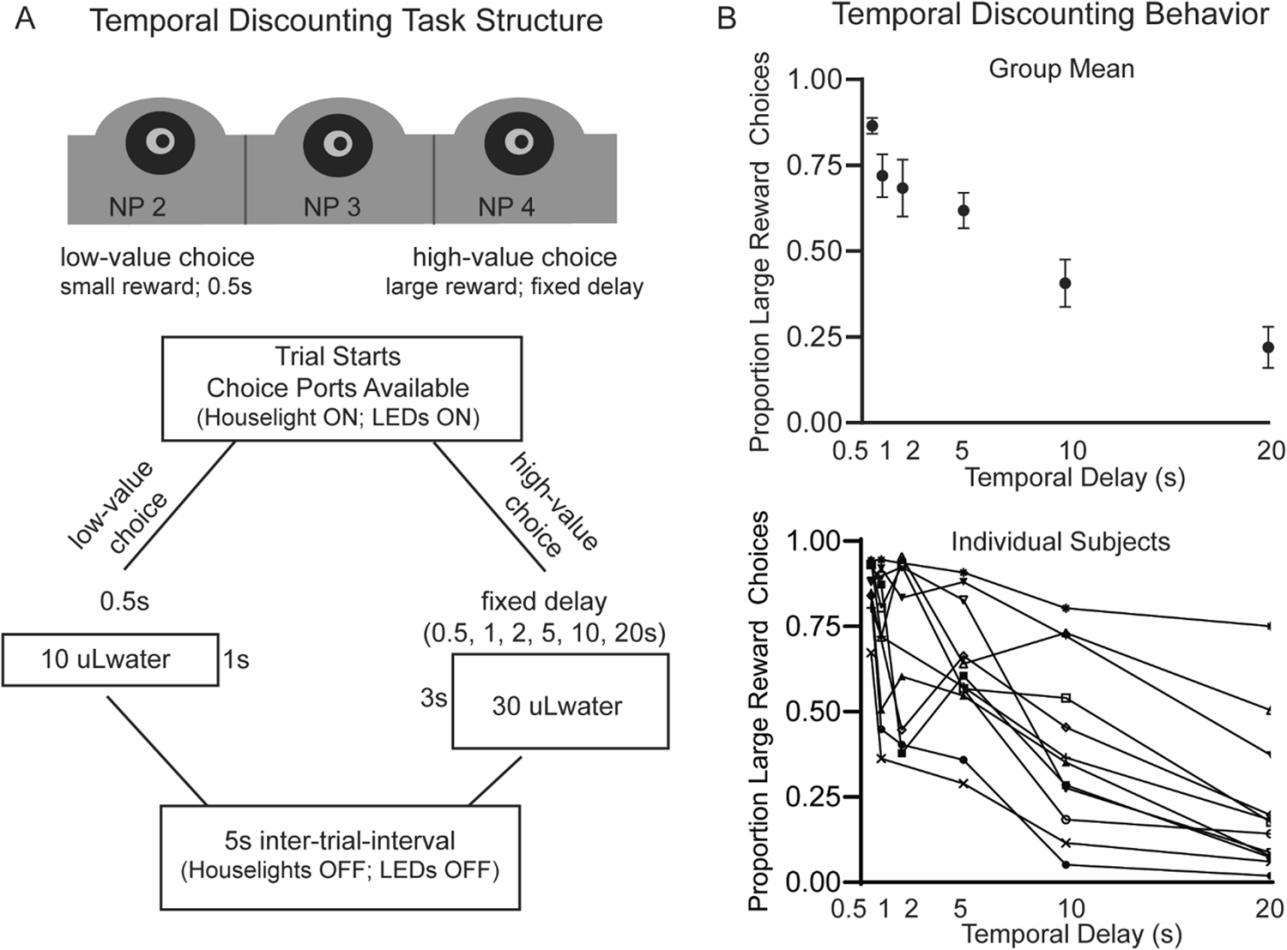
**Behavior on the temporal discounting task. A** Our version of the temporal discounting task centers around the subject selecting between a low-value choice (small reward delivered after 0.5 s) and a high-value choice (large reward delivered after a fixed delay). The large reward delay was fixed within a session, but changed from 0.5, 1, 2, 5, 10, and 20 s between sessions. Water reward was delivered from the middle noseport (NP3) at a rate of 10 µl/s. The small reward was 10 µl, and the large reward was 30 µl. **B** The group mean shows a significant effect of delay on percent of large reward choices within a session (*p* < 0.001; top panel). Subjects discount the large reward as its temporal delay increases (mean and SEM). There is a significant difference between individual subjects (*p* < 0.001), but no subject x delay interaction (*p* = 0.897; bottom panel). Full statistics are reported in Table S1

This experiment evaluated neural activity during reward-feedback modulated by reward magnitude and delay on a temporal discounting task. Animals were given the choice of a small reward (10 µl of water) delivered with a fixed delay of 0.5 s after response or a large reward (30 µl of water) delivered at a fixed delay that changed between sessions (0.5 s, 1 s, 2 s, 5 s, 10 s, 20 s) (Fig. 1A). To allow for greater numbers of trials necessary for robust electrophysiological analysis, delays on the large reward condition were kept constant throughout each session but varied across sessions. Results are based on 148 behavioral sessions (124 electrophysiology sessions) from 12 rats.

As expected, based on previous research, there was a main effect of delay on the proportion of large reward choices (F_(5,82)_ = 18.81, *p* =  < *0.001*) (Fig. 1B; top panel; Table S1). Animals’ preference shifted from large reward to small reward as the delay to reward increased. When delays were the same (0.5 s), animals strongly preferred the large reward (86.53 ± 7.60%). When the large reward followed a 20 s delay, rats only selected it on 21.99 ± 20.83% of trials, showing a clear preference for the immediate, small reward. There also was a main effect of subject (F_(11,82)_ = 4.60, *p* ≤ 0.001) (Fig. 1B; bottom panel; Table S1). Individual differences emerge in the average rate of discounting across delays, but there is no delay x subject interaction (*p* = 0.897).

#### Activity at Beta-Frequencies Reflects Reward Magnitude and Delay

**Fig. 2:**
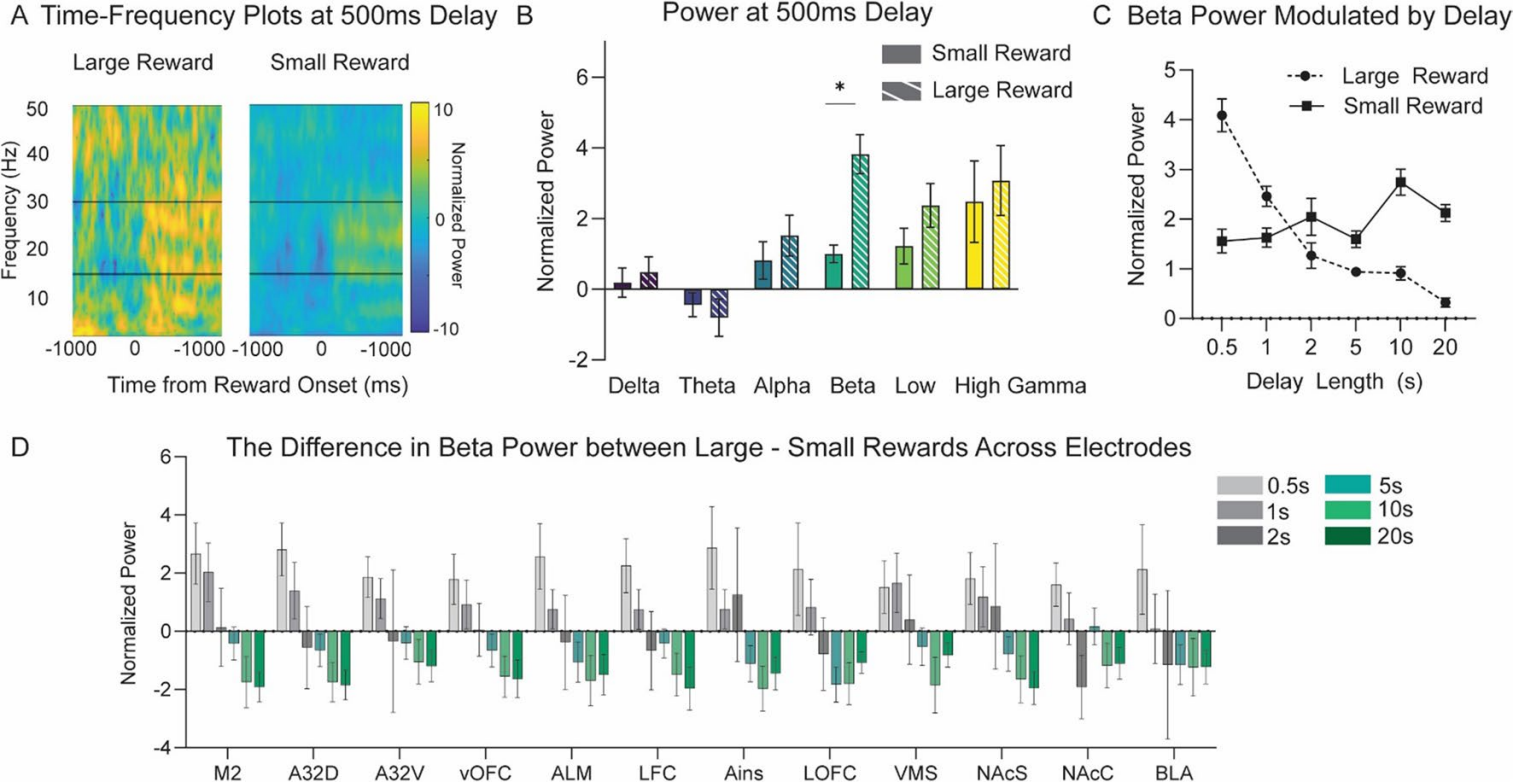
**Local field potential activity during the temporal discounting task. A** Time–frequency plots of lateral orbitofrontal cortex (lOFC) activity during large and small rewards when the delay for both is 500 ms from response is shown from one exemplar session. Activity is time-locked to reward onset (0 ms) and normalized power for large and small rewards is identically scaled. We see heightened beta-frequency (15–30 Hz) activity following either reward that is greater for the large reward choice. **B** LOFC power (normalized to baseline) at each canonical frequency band during the first second of reward delivery. Beta power is greater for large rewards (*p* < 0.006) when delays are equal (500 ms). The mean and SEM of LOFC power on small (solid) and large (striped) reward choices are shown. **C** Beta power modulates activity coinciding with temporal delay of the large reward. There is a significant interaction between delay and trial type (*p* < 0.001), such that lOFC beta power following the large reward choice decreases as the temporal delay to reward increases. Mean and SEM of lOFC power on large (dashed) and small reward (solid) are shown at each large reward delay. **D** Beta power is greater for large reward at short delays and greater for small reward at long delays at all 12 electrode locations (no effect of electrode; *p* = 0.063). Mean and SEM of the contrast of beta power for large-small reward. Abbreviations are listed in Table 1. Full statistics are reported in Table S2

First, to identify frequency bands of interest during the reward-feedback period, we analyzed normalized power in the lOFC electrode—a cardinal reward processing brain region (Dalley et al., 2004; Winstanley et al., 2004; Schoenbaum et al., 2009; Dalton et al., 2016; Constantinople et al., 2019; Wassum, 2022). We hypothesized that a putative value signal would have greater power for the large (30 µl) compared with the small (10 µl) reward when delays were equal (0.5 s for both). The time–frequency plots suggested elevated lOFC activity at beta (15–30 Hz) frequencies during reward outcome that was greater following large reward (Fig. 2A; one exemplar session).

To quantify the patten in the time–frequency plots and average across subjects and sessions, we used a linear mixed model that helps to account for variance in subject/sessions. We measured base-line normalized lOFC power one second following reward delivery across canonical frequencies (delta: 1–4 Hz; theta: 4–8 Hz; alpha: 8–12 Hz; beta: 15–30 Hz; low gamma: 30–50 Hz; and high gamma: 50–70 Hz) and between trial types (small or large reward). We analyzed only the first second of activity post-reward to ensure that, for both trial types, animals were receiving the same quantity of reward. Large reward choices result in 3 s (30 µl) of water, but small reward is only 1 s (10 µl) of water. To account for this difference, we analyzed only the first second of reward delivery, which was presumed to be equivalent for both trial types. When delays for each choice were equal (500 ms), there was a main effect of trial type (F_(1,164.43)_ = 5.54, *p* = 0.020), a main effect of frequency (F_(5,52.82)_ = 2.90, *p* = 0.022), and no interaction (*p* = 0.274) (Fig. 2B; Table S2). Subject contributed to 28% of variance in the model and session to only 0.2% of variance. Post-hoc tests (Bonferroni corrected) revealed that only beta frequencies had a significant difference between large and small reward trials (t_(34)_ = 2.14, *p* = 0.006), suggesting that it had the largest contribution to the main effect of trial type observed (Fig. 2B). The difference in baseline normalized beta power between the trial types was 5.19 ± 1.78.

Other frequency bands (gamma) showed a generic increase for reward, but only beta-frequencies were selectively elevated for large rewards. Therefore, we focused subsequent analyses on this frequency band. We next asked whether reward-locked beta power was sensitive to temporal delays of reward. We hypothesized the following: if beta-power only reflects the objective magnitude of the reward, then it would always be greater for the large reward regardless of delay. By contrast, if beta power reflects the reward value based on both magnitude and temporal delay, then we predicted it would be reduced for the large reward in concordance with increasing temporal delay. We also extended this analysis to 12 putative reward-related electrodes to determine if beta power during reward processing was unique to lOFC or seen broadly throughout the cortico-striatal reward network. To perform this, we used a second linear mixed-model to measure the effect of trial type (small or large reward), delay (0.5, 1, 2, 5, 10, 20 s), electrode (12 electrodes) and their interactions on reward outcome beta power. We found a main effect of delay (F_(5,2544.84)_ = 22.52, *p* < 0.001) and an interaction between delay x trial type (F_(5,2550.09)_ = 27.60, *p* < 0.001) (Table S2). First looking at the lOFC electrode to explain this interaction, we found that beta power on large reward trials decayed as the temporal delay increased (Fig. 2C). Beta power on small reward trials does not substantially change (or, if anything, slightly increases with increased delays) (Fig. 2C). Post-hoc tests (Bonferroni corrected) for the lOFC electrode show that at short delays (0.5 s, 1 s) there was greater beta activity for large reward trials (0.5-s delay, *p* < 0.001; 1-s delay, *p* = 0.014). At a moderate delay (2 s), there was no difference in power between trial types (*p* = 0.192). With long delays (5 s, 10 s, 20 s), there was greater beta power on small reward trials (5-s delay, *p* = 0.046; 10-s delay *p* < 0.001; 20-s delay, *p* < 0.001). This similar trend was observed at other electrode sites. Across the 12 putative reward regions (M2, A32D, A32V, vOFC, ALM, LFC, Ains, lOFC, VMS, NAcS, NAcC, BLA; Table 1), there was no significant difference in beta power between electrodes (*p* = 0.063) (Fig. 2D) or a significant electrode x delay x trial interaction (*p* = 1.00) (Table S2). On all electrodes, beta power at short delays was greater for large reward, and beta power at long delays was greater for small reward. Thus, beta power seems to reflect a subjective value estimate (opposed to an objective magnitude signal) that is dispersed broadly across areas of the cortico-striatal reward network.

#### **Network-Connectivity Linked with High-Value Choice**

**Fig. 3:**
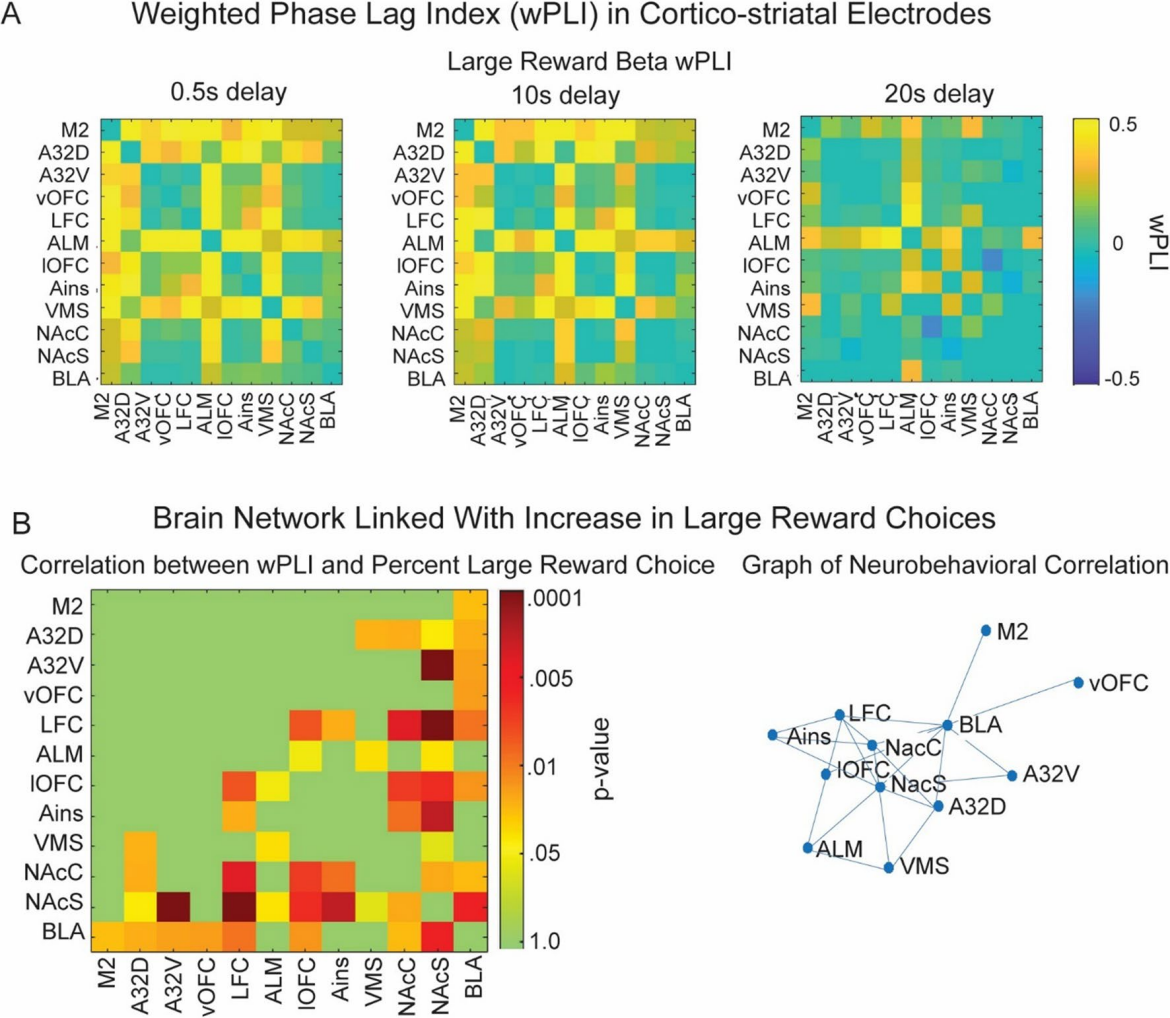
**Beta power in a reward-related network linked with large reward choice. A** Weighted phase-lagged index (wPLI), a measure of connectivity, is significantly positive between multiple electrode locations at beta frequencies during large reward choice including regions of prefrontal cortex, orbitofrontal cortex, and ventral striatum. Connectivity between these regions is the most robust (yellow) when the large reward temporal delay is short (0.5 s) and diminishes as the temporal delay increases (20 s). **B** We used a GLM to examine the relationship between the percent of large reward choice within a session and the beta power wPLI during the first second of reward delivery. The nonthresholded *p*-value from the GLM is shown for each electrode pair. *P*-values do not provide information about the strength of correlation for each pair. We only observe positive relationships between electrode sites. The *p*-values and beta values for the GLM are included in Table 2. The network graph highlights the significant nodes of beta-frequency wPLI during large rewards anchored around nucleus accumbens/amygdala and lOFC. Abbreviations included in Table 1

To better understand whether beta oscillations during reward outcome reflects a “network-wide” phenomenon, we used a measure of connectivity termed weighted phase-lagged index (wPLI). While no method is perfectly robust to all noise/volume conduction issues, this method is somewhat more robust than many others (Vinck et al., 2011). We first analyzed reward-locked wPLI at beta-frequencies across the first second post-reward on the high-reward trials at each delay (Fig. 3A shows mean wPLI estimated across three distinct delays). Given the high number of variables (connections between 12 electrode sites at 5 different delays), we focused our statistical analysis on one simple question: is there a relationship between beta-frequency wPLI and high-value choice behavior? To calculate this, we used a generalized linear model to estimate slope (beta-values), with percent of large reward choice as the independent variable and pair-wise estimates of the wPLI during the first second of reward as the dependent variable across sessions. A beta-value and *p* value from the model (percent large reward choice x wPLI) was calculated for each electrode pair followed by FDR-correction across the connectivity matrix (Fig. 3B; Table 2). We found a significant positive relationship (*p* ≤ 0.05) between wPLI strength and selection of the large reward for several brain regions that includes lOFC, prefrontal cortex, ventral striatum/nucleus accumbens (Fig. 3B; *p* value does not provide any information about the strength of the correlation). The right panel illustrates the pair-wise connections with a significant relationship between wPLI and large reward choice. Nucleus accumbens, lOFC, and amygdala are at the center of the network, with the most numerous significant correlations with other electrodes. We did not observe any significant negative relationships (where greater wPLI on the large reward trial was linked with less choice for large reward). Thus, in general and consistent with our findings from LFP power, greater beta-frequency connectivity between cortical and striatal regions (as measured using the wPLI) during high-value rewards was associated with greater selection of the large reward choice.

**Table 2:** GLM statistics

GLM *p*-values	M2	A32D	A32V	vOFC	LFC	ALM	lOFC	Ains	VMS	NacC	NacS	BLA
M2	1.000	0.060	1.000	1.000	1.000	1.000	1.000	1.000	1.000	0.099	0.050	0.021
A32D	0.060	1.000	1.000	0.071	1.000	1.000	1.000	1.000	0.006	0.000	0.021	0.006
A32V	1.000	1.000	1.000	1.000	1.000	0.076	1.000	1.000	0.067	1.000	0.000	0.034
vOFC	1.000	0.071	1.000	1.000	1.000	1.000	1.000	0.054	0.060	0.076	1.000	0.004
LFC	1.000	1.000	1.000	1.000	1.000	1.000	0.004	0.007	1.000	0.001	0.001	0.009
ALM	1.000	1.000	0.076	1.000	1.000	1.000	0.007	0.060	0.023	0.067	0.005	1.000
lOFC	1.000	1.000	1.000	1.000	0.004	0.007	1.000	0.081	0.083	0.000	0.048	0.016
Ains	1.000	1.000	1.000	0.054	0.007	0.060	0.081	1.000	1.000	0.021	0.000	1.000
VMS	1.000	0.006	0.067	0.060	1.000	0.023	0.083	1.000	1.000	1.000	0.028	0.067
NacC	0.099	0.000	1.000	0.076	0.001	0.067	0.000	0.021	1.000	1.000	0.016	0.009
NacS	0.050	0.021	0.000	1.000	0.001	0.005	0.048	0.000	0.028	0.016	1.000	0.006
BLA	0.021	0.006	0.034	0.004	0.009	1.000	0.016	1.000	0.067	0.009	0.006	1.000
GLM beta values	M2	A32D	A32V	vOFC	LFC	ALM	lOFC	Ains	VMS	NacC	NacS	BLA
M2	0.000	0.000	0.000	0.000	0.000	0.000	0.000	0.000	0.000	0.000	0.000	0.741
A32D	0.000	0.000	0.000	0.000	0.000	0.000	0.000	0.000	0.785	0.810	0.573	0.810
A32V	0.000	0.000	0.000	0.000	0.000	0.000	0.000	0.000	0.000	0.000	2.825	0.850
vOFC	0.000	0.000	0.000	0.000	0.000	0.000	0.000	0.000	0.000	0.000	0.000	0.868
LFC	0.000	0.000	0.000	0.000	0.000	0.000	1.171	0.798	0.000	1.602	2.346	1.041
ALM	0.000	0.000	0.000	0.000	0.000	0.000	0.478	0.000	0.621	0.000	0.594	0.000
lOFC	0.000	0.000	0.000	0.000	1.171	0.478	0.000	0.000	0.000	1.296	1.364	0.906
Ains	0.000	0.000	0.000	0.000	0.798	0.000	0.000	0.000	0.000	1.047	1.744	0.000
VMS	0.000	0.785	0.000	0.000	0.000	0.621	0.000	0.000	0.000	0.000	0.405	0.000
NacC	0.000	0.810	0.000	0.000	1.602	0.000	1.296	1.047	0.000	0.000	0.813	0.753
NacS	0.000	0.573	2.825	0.000	2.346	0.594	1.364	1.744	0.405	0.813	0.000	1.441
BLA	0.741	0.810	0.850	0.868	1.041	0.000	0.906	0.000	0.000	0.753	1.441	0.000

#### **Computational Model Estimates Subjective Value on the Temporal Delay Task**

Our data above suggest that beta-oscillations reflect some aspect of temporal discounting behavior. To offer one interpretation of how this beta signal may reflect behavior, we used computational modeling to estimate the subjective value across different delays. Subjective value describes the individual preference to choose large versus small reward based on objective factors of reward magnitude and delay and internal factors, such as motivation. In the computational model, subjective value of the action selected is related to the magnitude of reward (10 or 30 µl) and the delay to reward delivery (0, 1, 2, 5, 10, or 20 s for the large reward; 0 s for the small reward). Previous work in both animals and humans have explored various fits to temporal discounting curves, including exponential and hyperbolic (Kable & Glimcher, 2007; Vanderveldt et al., 2016). We fit models that utilized a linear, hyperbolic, or exponential discount function to our behavioral data. We found that the model using an exponential discount function was associated with the lowest AIC value (Exponential = 2387.06; Hyperbolic = 2452.39; Linear = 2868.35).

**Fig. 4:**
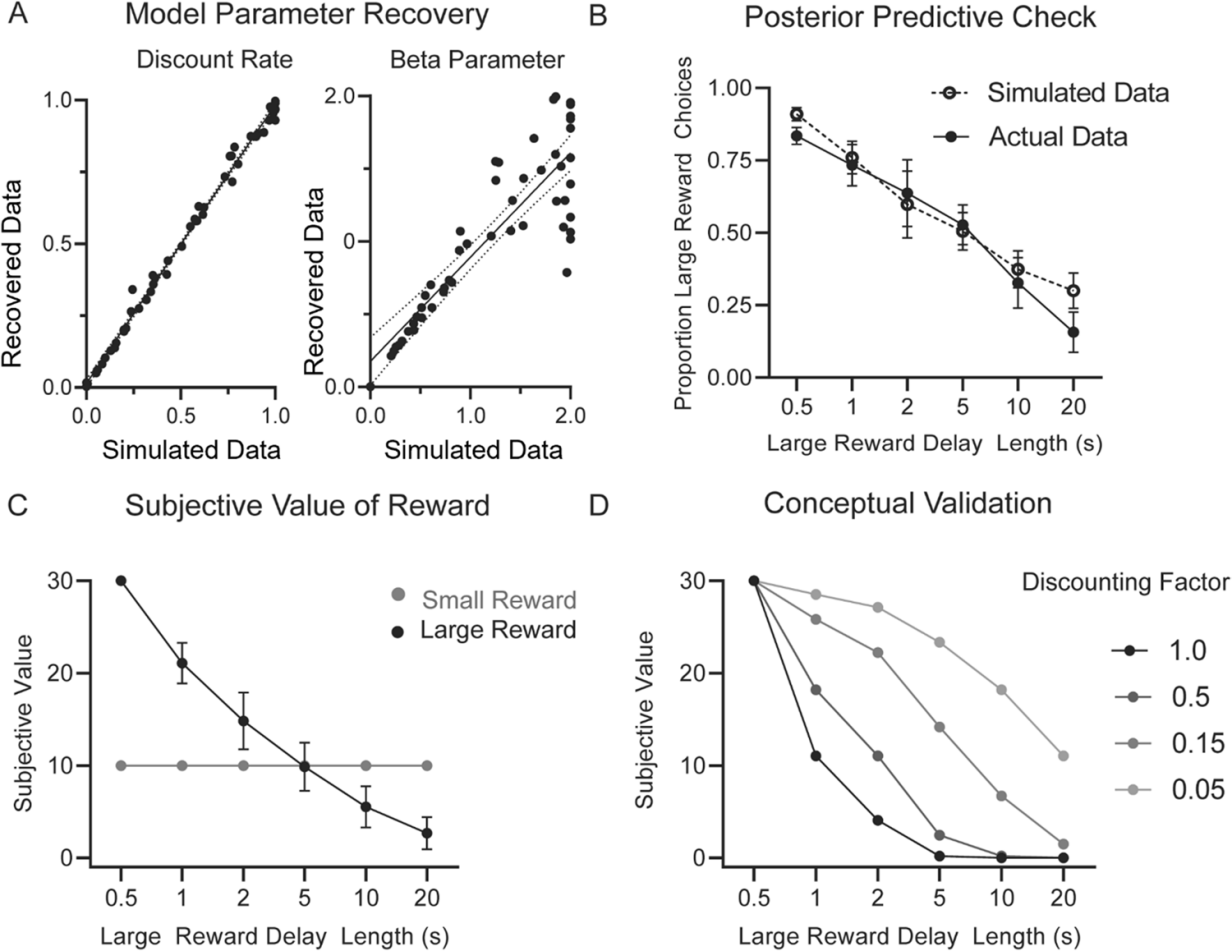
**Computational model to define subjective value on the temporal discounting task. A** The parameter recovery estimates show that the simulated and recovered discounting rates are highly related (R^2^ = .994). Likewise, the estimated and actual beta values are highly related (R^2^ = .755), thus confirming that the parameter values can be reliably estimated. **B** The posterior predictive check shows no difference between the proportion of large reward choices in the simulated (exponential model; dashed line) and actual rodent data (solid line) (*p* = 0.358). **C** Subjective value of the large reward starts at 30 (reflecting 30-µl reward amount) and decreases exponentially as the temporal delay increases. The subjective value of the small reward starts at 10 (reflecting 10-µl reward amount) and does not change because its delay is fixed at 0.5 s while the large reward is increasingly delayed. **D** The simulation validation shows that as the discount rate of our model increased, the subjective value of the large reward decreases at a faster rate

To validate our computational model, we first ensured that the parameter values estimated for each subject were recoverable (Tranter et al., 2023; Wilson & Collins, 2019). We simulated performance in the delayed discounting task 50 times using known parameter values and then fitted the exponential model to the simulated behavior (Fig. 4A). A positive correlation between the simulated and recovered values was evident for the discount rate (R^2^ = 0.994, β = 0.969) and the beta parameter (R^2^ = 0.755, β = 0.717), demonstrating that our model could accurately estimate the free parameter values. Next, we simulated task performance using the model parameters we estimated from the actual rodent performance. This posterior predictive check demonstrated that the proportion of large reward choices for the simulated data was comparable to that of the actual rodent data (two-way ANOVA main effect of group (F_(1,109)_ = 0.85, *p* = 0.358; Fig. 4B), thereby confirming that our exponential model could capture the key behavioral components associated with task performance.

Using this model, we found that subjective value of the large reward progressively decreased as the delay associated with obtaining it increased (Fig. 4C). When the delay was < 5 s, the large reward had a greater subjective value than the small reward; however, when the delay exceeded 5 s, the subjective value for the large reward was smaller than the small reward. This change in the relative subjective value associated with either option may explain the shift to prefer small reward choices observed behaviorally (Figs. 1B and 4B).

Finally, to provide conceptual validation of our model, we simulated performance using an agent with varying discount rates (but keeping the beta parameter fixed; 0.1). As expected, when the discount rate is larger, the subjective value of the large reward decays more rapidly as the delay increases (Fig. 4D).

#### **Beta Power is Related to Subjective Value as Defined by the Computational Model**

**Fig. 5:**
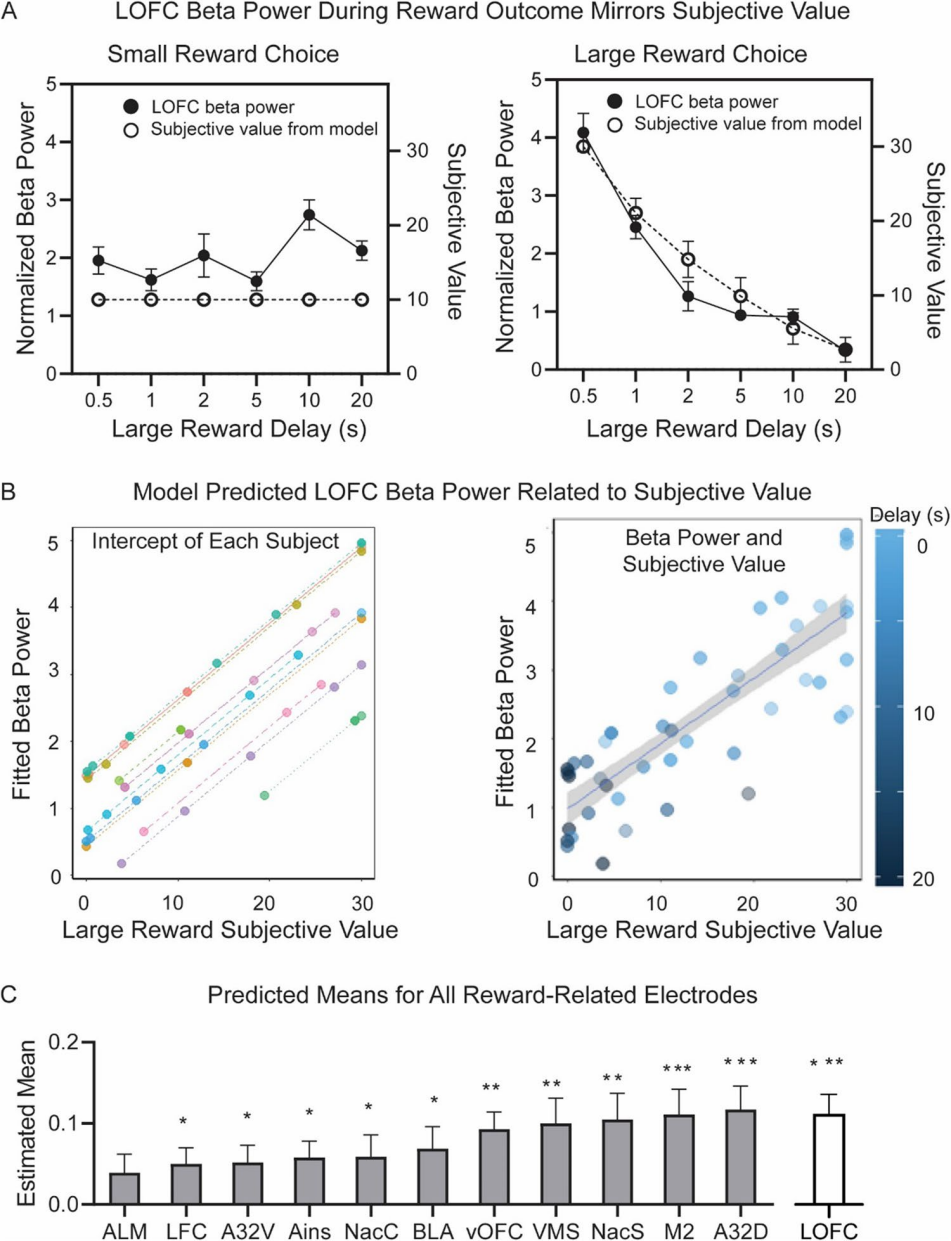
**Subjective value is related to large reward beta power**. **A** LOFC Beta power during the first second of reward outcome is related to subjective value as defined by our computational model. On small reward choice, neither beta power (filled circle, solid line) nor subjective value (open circle, dashed line) is modulated by large reward delay. On large reward choice, both beta power and subjective value decay at a similar rate as temporal delay of the large reward increases. **B** We used a linear mixed model to quantify the observed relationship between subjective value and large reward beta power with subject as a random factor. Each subject has a positive relationship between fitted LOFC beta power and subjective value of the large reward, but the intercept for each subject differed (left panel). Concatenated across all subjects, at long delays (dark circles) both the beta power and subjective value are small, whereas at short delays (light circles) power and subjective value are high (right panel). **C** The predicted means (and SEM) from the model are shown in rank-order for all 12 electrodes. LOFC data are included again as a comparison. Only ALM was not significant. *p < 0.05; **p < 0.001; ***p < 0.0001

Next, we correlated reward outcome beta power on the lOFC electrode with subjective value as defined by our computational model. Upon observation alone, neither beta power nor subjective value was modulated by temporal delay on small reward choice, but on large reward choice beta power decayed at a similar rate as subjective value (Fig. 5A).

We used a linear mixed-effects model that included lOFC beta as the dependent variable, the subjective value of the large reward as the independent variable, and subject as a random factor to quantify this observation. A significant relationship between lOFC beta power and value was evident (β = 0.11 ± 0.024, *p* < 0.001) indicating that as lOFC beta power increased, so did the subjective value. To illustrate this relationship further, we extracted the predicted beta power values from the fitted model (Fig. 5B). The predicted beta frequency values from the linear mixed model are related to the large reward subjective value. At long delays, both the large reward subjective value and the beta frequency power are smaller. At short delays, subjective value and beta power are greater. The predicted values from the linear mixed model show the slope between beta power and large reward subjective value separately for each subject (Fig. 5B, right panel). Finally, we expanded this analysis to include the other 12 electrodes. The estimated marginal mean from the linear mixed model for each electrode is plotted in rank order (Fig. 5C). All electrodes were significant except ALM.

To further validate our model and explore the relationship between large reward subjective value and delay length, we repeated the above using two alternative models; one with large reward delay length as the independent factor and one with both delay length and subjective value as factors. All models had significant effects on lOFC beta power, but our first model using large reward subjective value as the independent factor accounted for more variance in lOFC beta power (93.69%) and had the lowest AIC and BIC scores (391.3, 400.19) and therefore was determined to be the best model to use.

#### **Beta frequency stimulation modulates temporal discounting choice**

The goal of the stimulation experiment was to test the hypothesis that beta power in the cortico-striatal network, during reward outcome, represents choice value. We predicted that providing beta stimulation on the large reward condition would increase the frequency of that choice, despite the temporal delay. The experiment is included as preliminary supporting evidence, because it is consistent with our electrophysiology observations but lacks important controls, such as stimulation at other frequencies, different delay conditions, replication of sites and parameters. We used an “on-demand” electrical stimulation approach in six rats, delivering beta frequency (20 Hz) stimulation during the reward outcome period (3 s) of the high-value choice. The electrical stimulation parameters (current, electrode location) were adjusted for each subject (Table S3). Notably, to test our hypothesis regarding the fact that this was a distributed signal, we provided stimulation at different sites in each animal. Of all sites within the putative reward network, the electrode with the lowest impedance was selected. Sites included ALM, A32D, A32V, Ains, vOFC, and VMS (Table 1). Amplitude was adjusted according to the impedance measure but ranged from 35–80 µA. The temporal delay for the large reward was different for each subject (2 s, 5 s, or 10 s) but remained consistent throughout all stimulation sessions for each animal. The delay for the small reward was fixed at 0.5 s.

**Fig. 6:**
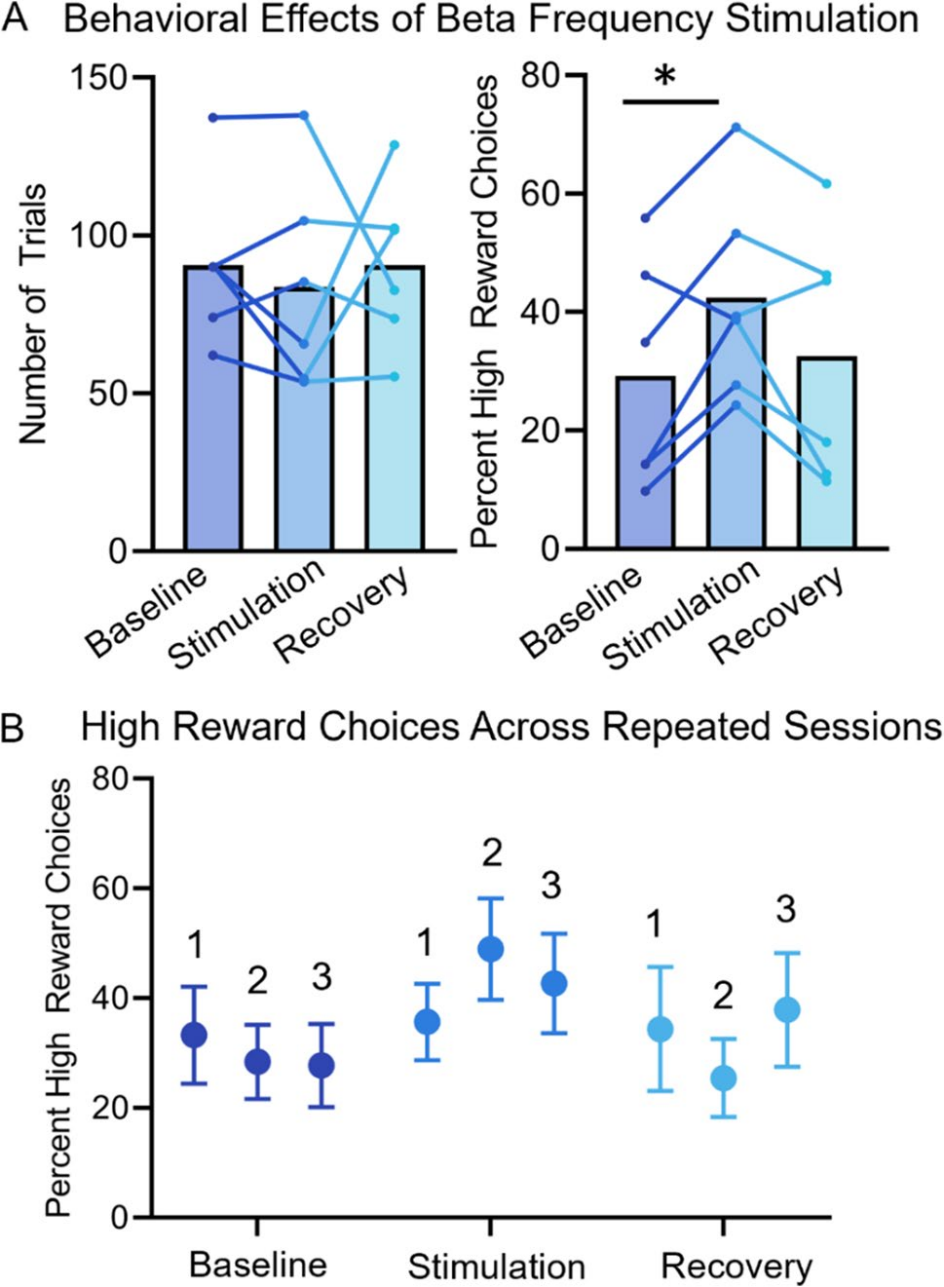
**On-demand beta frequency stimulation. A** The behavioral effects of beta frequency stimulation during large reward outcome in a temporal discounting task in six rats. Stimulation parameters are provided in Table S3. Behavioral parameters (number of trials and percent of high-value choice) are measured across three baseline days (no stimulation), three stimulation days, and three recovery days (no stimulation). Beta frequency stimulation had no effect on the number of trials (*p* = 0.293 but did increase the percent of high-value choices compared to baseline (*p* = 0.030). Lines represent the mean for each subject. B) We observe a significant day x stimulation condition effect (*p* = 0.029), driven by significant differences between day 2 stimulation and day 2 baseline (*p* = 0.017) and day 2 recovery (*p* = .021), suggesting that stimulation had a maximal effect after repeated sessions

We used a two-way repeated measures ANOVA to test the within-subjects effects of condition (baseline, stimulation, recovery) and session (3 of each condition) on the dependent measures of percent high-value choices and number of trials. The number of trials was not significantly different across conditions (F_(2,10)_ = 1.39, *p* = 0.293) or days (F_(2,10)_ = 0.42, *p* = 0.645) (Fig. 6). However, there was a significant condition x day interaction (F_(4,20)_ = 5.55, *p* = 0.029) for the percent of high-value choices (Fig. 6). Beta frequency stimulation increased the proportion of high-value choices within a session compared with baseline (F_(1,5)_ = 8.99, *p* = 0.030). On average stimulation increased high-value responding (from a mean of 29.25% to 42.43%). Post-hoc tests showed that the interaction between condition x day was driven by significant differences between day 2 baseline versus stimulation (*p* = 0.017, mean difference = 20.59) and day 2 stimulation versus recovery (*p* = 0.021, mean difference = 23.55) (Fig. 6). Therefore, stimulation had its maximal effect during the second consecutive session. Generally, the proportion of high-value choices was similar to baseline on recovery days following stimulation. Individual differences in results may be owing to impedance, current, electrode site, or behavioral parameters and will need to be standardized in future tests. However, notably, we show that stimulating beta frequencies on striatal, insular, prefrontal, and orbitofrontal sites during reward outcome can directly influence value-based decision making suggesting a distributed signal. Further studies will be needed to better understand the specificity of these results according to frequency and site (i.e., further testing at “control” electrode sites”).

## Discussion

In humans and animals, temporal discounting tasks have been used to classify impulsivity and maladaptive behavior associated with neuropsychiatric disorders, including schizophrenia, depression, attention deficit hyperactivity disorder, and substance use (Mitchell, 2019; Story et al., 2014). Defining a brain signature that reflects reward processing on this task could help to uncover the mechanisms behind value-based decision making and may offer a potential therapeutic target to treat such disorders. In this study, we used LFP recordings in rodents to identify a brain state associated with temporal discounting behavior. We identified cortico-striatal beta oscillations that reflect both reward magnitude and temporal delay and correlate with subjective value as defined by a computational model. Previous research posits that the cortico-striatal network is important for reward valuation (especially during temporal discounting) (Ballard & Knutson, 2009; Bayer & Glimcher, 2005; Boettiger et al., 2007; Haber & Knutson, 2010; Pornpattananangkul & Nusslock, 2016; Snyder et al., 2020), and we believe that cortico-striatal-limbic beta oscillations during reward outcome may reflect this process.

In the temporal discounting task, rats choose between a small reward delivered with a fixed, short delay, or large reward that was given with fixed delays that varied across sessions. Animals were less likely to select the large reward with increased delays (Fig. 1). We measured normalized power across canonical frequency bands during the first second of reward-feedback. Because we were limited by the number of statistical comparisons (frequencies, delays, electrodes), we restricted our analyses to reward outcome (not trial onset, choice, or delay period), consistent with our motivation to examine neural markers of reward valuation. Even in well-trained animals, there is likely an updating that needs to occur after reward outcome based on objective measures of reward magnitude and delay to inform future decisions. Beta (15–30 Hz) frequency power during reward outcome tracked objective reward magnitude when delays were matched (0.5 s) and scaled dynamically as value as a byproduct of temporal delay (Fig. 2). Connectivity between BLA, ventral striatum and prefrontal/orbitofrontal cortex during the high-value reward was correlated with the proportion of large reward choices across delay conditions (Fig. 3). Next, we used a computational model to estimate subjective preference based off the observed behavior for each subject. In temporal discounting tasks, subjective preference (value) changes based on objective task variables (delay and reward magnitude). The model showed a positive relationship between beta frequency power and subjective value, offering one possible interpretation of beta activity in the cortico-striatal-limbic network might reflect behavior on this task (Figs. 4 and 5).

In a preliminary data set, we show that modulating beta-frequency (20 Hz) activity with electrical stimulation can bias behavior toward a larger, delayed reward. By applying “on-demand” stimulation (stimulation based on the rats’ behavior) during the reward-outcome period of large reward choices, we were able to increase the number of high reward choices made within a session (Fig. 6). Multiple cortical (prefrontal, orbitofrontal, insular) and striatal (ventral striatum) stimulation targets (Table S3) lead to significant changes in discounting behavior, consistent with our findings from the wPLI/connectivity analysis that suggest beta oscillations are a network-wide phenomenon. These results support our observation that beta oscillations during reward outcome are correlated with subjective value but should be interpreted with caution because of these several limitations: stimulation was not tested at other frequencies; results are limited to only one delay condition; there is a lack of replication in stimulation parameters and sites; and there is no control to determine if stimulation is rewarding. Moreover, in this preliminary study, LFP was not paired with electrical stimulation; therefore, we cannot be certain that stimulation was enhancing (opposed to disrupting) oscillatory activity, although prior work shows that beta-frequency (15 Hz) deep-brain stimulation in rats does not suppress local (globus pallidus) or distal (dorsal striatum and motor cortex) oscillations (McCracken & Kiss, 2014).

We observe that beta oscillations in the cortico-striatal-limbic network change based on reward magnitude and delay. One interpretation, based on the computational model of behavior, is that beta oscillations reflect subjective reward value. Alternative explanations of our findings are that beta activity reflects: 1) a nonneural artifact time-locked with reward delivery (i.e., muscle/EMG-related contamination during reward consumption or electrical noise associated with reward delivery); or 2) is neurophysiological in nature but does not reflect rewards per se (e.g., time estimation or working memory). To elaborate more on this idea, beta-oscillations have been well-characterized within motor cortex (Witham et al., 2007; Kilavik et al., 2012; Feingold et al., 2015; Khanna & Carmena, 2017; Luhmann et al., 2021) and dorsolateral striatum (Feingold et al., 2015; Jenkinson & Brown, 2011; Luhmann et al., 2021; Schwerdt et al., 2020) and tend to be largest pre/post-movement but are classically suppressed during movement (Hammond et al., 2007; Engel & Fries, 2010; Kilavik et al., 2012; Khanna & Carmena, 2017). Thus, one explanation for our findings is that increased beta power reflects motor inhibition that might occur during reward consumption. However, there are several aspects of our data that are not consistent with a motor-inhibition explanation. First, sensorimotor beta-oscillations, as previously described, are localized within motor and dorsal striatum, whereas we observe oscillations in ventral cortical orbitofrontal cortex, insula, and striatal brain regions. Second, we observe greater beta power selectively for large reward choices when delays (and thus noise/motor activity) would be most likely to be balanced (and even using the same reward 1-s post-reward delivery window). We unfortunately did not collect the data we need (high-frequency video of licks/motor behavior) to properly quantify consummatory behavior. This requires further research for clarification. Similarly, noise/muscle artifacts would not obviously lead to phase-lagged differences between distinct nodes of the reward network as we observed (difference in wPLI correlating with choice) and that again were strongest in nonmotor parts of PFC. It also is possible that beta power represents time estimation or working memory related to the reward delay. Several studies implicate beta power in working memory maintenance and clear-out (Chen & Huang, 2016; Miller et al., 2018; Schmidt et al., 2019; Spitzer & Haegens, 2017b). Beta power increases with working memory load (Chen & Huang, 2016) and is heightened at the end of a trial which is speculated to represent memory clear-out (Schmidt et al., 2019). If the beta signal is a time estimation or working memory signal, it is not clear why we see greater beta power for large reward when the delays are equal (500 ms) or why beta power is decreased following longer delays. Because the goal of this paper was to specifically examine reward-related activity, we are missing timeframes of interest (such as response-locked data) that may provide a more holistic view of beta during active memory maintenance, memory clear-out, and reward anticipation. We believe that the theory most consistent with the totality of our data is that beta oscillations integrate information about objective task variables (reward magnitude and temporal delay) to signal subjective preference for reward.

Growing research has identified beta-oscillations outside of sensorimotor networks related to attention (Schmidt et al., 2019; Shin et al., 2017), top-down processing (Buschman & Miller, 2007; Engel & Fries, 2010), working memory (Marco-Pallarés et al., 2015; Schmidt et al., 2019; Siegel et al., 2009; Spitzer & Haegens, 2017a), effort (Hoy et al. 2024), and outcome evaluation (Pesaran et al., 2008; Torrecillos et al., 2015). Consistent with our findings, beta-oscillations during reward feedback have previously been observed in humans and animals. EEG and MEG measures in humans find beta oscillations during positive-valence reward within cortico-striatal circuits that are sensitive to reward valence, magnitude, and predict subsequent choice (Hoy et al., 2024; Cohen et al., 2007; Marco-Pallares et al., 2008; HajiHosseini & Holroyd, 2015; Marco-Pallarés et al., 2015; Zavala et al., 2018; Patai et al., 2022). Most relevant to our study, data from humans on a temporal discounting task showed that feedback-locked theta and beta activity measured with EEG was associated with a preference for larger, delayed rewards (Pornpattananangkul & Nusslock, 2016). Theta activity is thought to be a product of cognitive-control processes integrating reward outcome information and is reliably stronger for bad-performance feedback and predicts behavioral adjustment on the subsequent trials (Cohen et al., 2011). Conversely, beta activity is consistently related to positive reward feedback (Cohen et al., 2007; HajiHosseini & Holroyd, 2015; Marco-Pallares et al., 2008; Marco-Pallarés et al., 2015; Patai et al., 2022; Pornpattananangkul & Nusslock, 2016; Zavala et al., 2018). Both ventral striatum and LOFC activity measured with fMRI also are associated with a preference for larger, delayed rewards (Ballard & Knutson, 2009; Boettiger et al., 2007). Reward processing signals in these regions may predict individual differences in the tendency to wait for larger rewards over small, immediate rewards, important in the context of relating reward sensitivity with high-risk behaviors seen in many neuropsychiatric disorders.

Similarly, increased beta power in cortico-striatal regions has been observed in rodents approaching reward locations (Howe et al., 2011; Samson et al., 2017) that was modulated by reward magnitude (Samson et al., 2017) and stabilized with task experience (Howe et al., 2011; van der Meer & Redish, 2009). Recently, it was observed that during a reward discrimination task, increased beta power 100–200 ms after reward-feedback in the anterior cingulate cortex and nucleus accumbens of rodents was correlated with response bias (Iturra‑Mena et al., 2023). The congruency with human EEG studies bolsters the translatability of using animal LFP to define brain-based biomarkers of reward processing.

Although we find a significant relationship between reward value and beta oscillations, how reward modulates beta frequency activity remains unclear. One hypothesis is that beta frequency activity during reward processing is linked with dopamine. Dopaminergic, “reward-prediction-error” (RPE) signals are related to value estimation, i.e., they are positively modulated by difference between expectation of reward and reward delivery (Schultz 1997; Bayer & Glimcher, 2005; HajiHosseini & Holroyd, 2015; Snyder et al., 2020). The feedback-related negativity ERP signal classically observed in humans is negative following positive reward and is thought to reflect dopamine transmission (Holroyd & Coles, 2002; Iturra-Mena et al., 2023; Walsh & Anderson, 2012); the opposite of our beta oscillatory signal. Previous research in humans explored the possibility of frontal beta-oscillations as an RPE signal but found that stimuli signaling expected rewards elicited more beta power than unexpected rewards; the inverse of an RPE (HajiHosseini & Holroyd, 2015). Thus, we believe our results are consistent with an inverse correlation between beta activity and the dopaminergic RPE signal. During temporal discounting paradigms, dopaminergic activity is greater for longer delay periods (Kobayashi & Schultz, 2008), whereas we observe reduced beta power during these longer delay periods. Interestingly, an inverse relationship between dopamine and beta-oscillations has been observed in motor cortex and dorsolateral striatum as well (Bayer & Glimcher, 2005; Schwerdt et al., 2020). Therefore, a similar relationship between beta-oscillations and dopamine may exist within ventral striatum and prefrontal cortex. The inverse of an RPE (the action value) is thought to reflect a cortico-striatal weight. During decision-making, cortico-striatal weights are strengthened or weakened based on RPE signals (Barnett et al., 2023; Vich et al., 2020). Thus, our marker of reward-related cortico-striatal beta oscillations may reflect this weight formation process, used to inform future decisions and is known to be impaired in cases of neuropsychiatric disorders, such as depression (Kumar et al., 2018).

Future analyses will need to investigate network-level connectivity at the level of single units or by using methods (such as calcium imaging) in which distinct networks can be targeted to determine whether beta-oscillations originate in brain areas, such as the striatum, or whether they are an emergent property of cortico-striatal networks, and the degree to which they are influenced by dopamine. Previous research suggests that focal versus long-range beta oscillations may operate at different frequencies to serve different functions (Bonaiuto et al., 2021; Seedat et al., 2020); thus, it will be important to define local processing with single unit investigations. Additionally, our results are mostly limited to male rats. We are now repeating this set of studies in a balanced cohort of male/females to understand whether these findings generalize across sexes and plan to expand the stimulation study with appropriate controls. We propose that beta-oscillations during reward-feedback may present a phenotype that can be used to identify disturbed reward-related processing deficits in psychiatric disorders. The beta oscillations that we observe appear in physiologically relevant time windows (reward outcome), making this a potential signature to influence trial-by-trial decision-making behavior. If these findings can be extended across reward-guided decision-making tasks, it will suggest that beta-oscillations can be utilized as a cross-species translational marker of value estimation that is linked to reward-guided behavior and could be used to predict reward sensitivity, risk-taking behavior, and impulsivity.

## Supplementary information

Below is the link to the electronic supplementary material.

Supplementary file1 (DOCX 2666 KB)

## Data Availability

The data included in the main text is available in a DANDI archiver repository (https://dandiarchive.org/dandiset/000952). Additional data, methodological details, and equipment will be made available upon request.

## Code availability

Associated analysis code will be publicly available to the corresponding author’s Github (https://github.com/mfrancoeur648).
