# Supplementary material for: Cortico-striatal beta oscillations as a reward-related signal
Source: Cogn Affect Behav Neurosci. 2024 Aug 15;24(5):839–59. doi: 10.3758/s13415-024-01208-6 (PMC11390840; doi:10.3758/s13415-024-01208-6)
Supplement: Supplementary file 1 — Supplementary file1 (DOCX 2666 KB) [file 13415_2024_1208_MOESM1_ESM.docx]

**Supplement**

**Cortico-striatal beta oscillations as a reward related signal**

**Running title:** (Beta oscillations represent reward value)

**M.F. Koloski** **^1,2^, S. Hulyalkar ^1,2^, S.A. Barnes^2^, J. Mishra ^2^, D.S. Ramanathan ^1,2^.**

1. Mental Health Service, VA San Diego Healthcare Syst., La Jolla, CA, 92161

2. Dept. of Psychiatry, UC San Diego, La Jolla, CA, 92093

**Corresponding author:** Miranda F. Koloski

Email: mfrancoeur@health.ucsd.edu

Phone: (603) 988-7516

**Conflict of Interest:** The authors declare no competing financial interests.


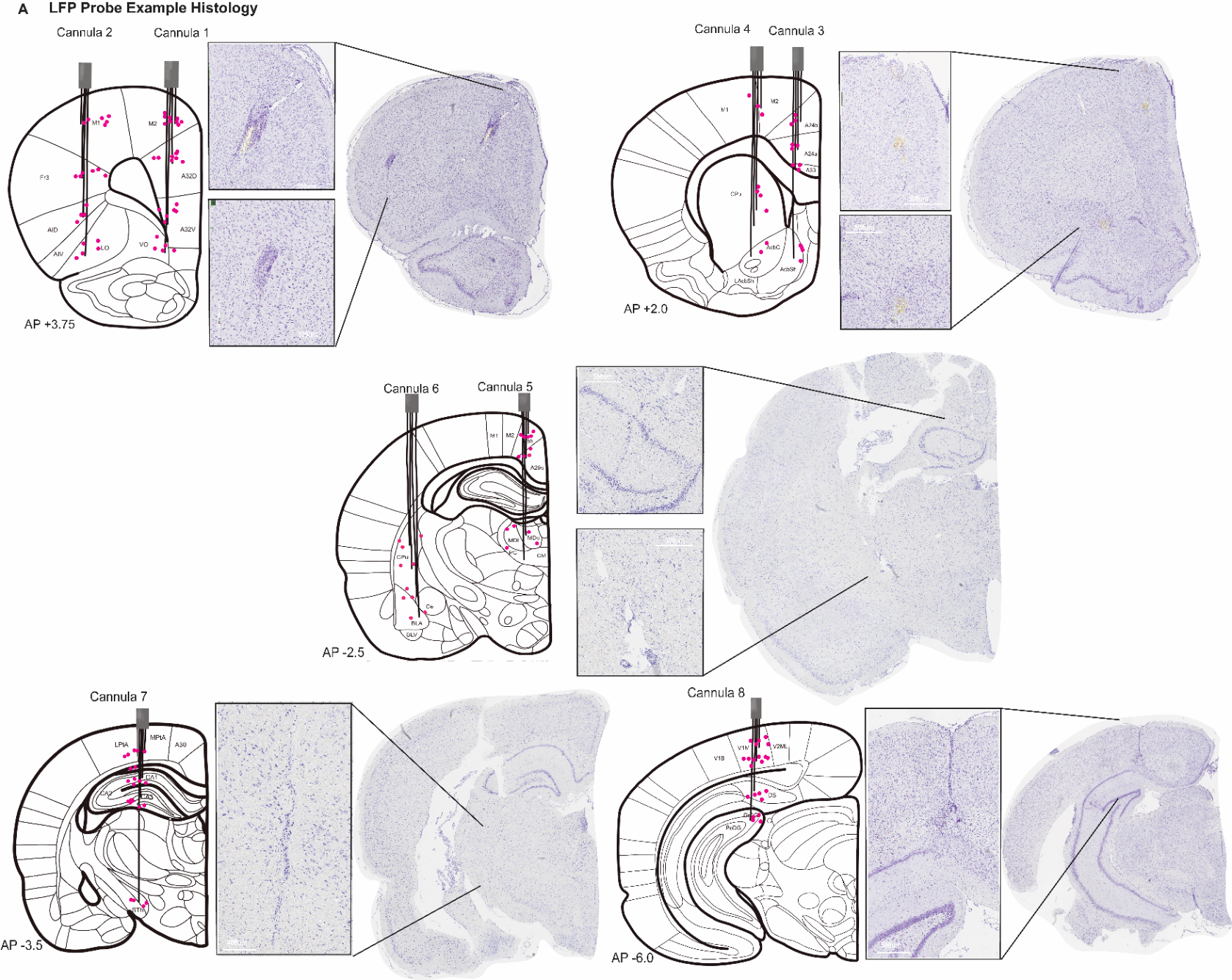


**B.**

| **AP** | **ML** | **DV** | **Target Area** |
| --- | --- | --- | --- |
| Cannula 1 | | | |
| 3.75 | 0.8 | 0.8 | Motor Area 2 |
|  |  | 3.2 | A32D |
|  |  | 4.8 | A32V |
|  |  | 5.8 | Ventral Orbitofrontal Cortex |
| Cannula 2 | | | |
| 3.75 | 3.2 | 1.0 | Anterolateral Motor Cortex |
|  |  | 3.6 | Lateral Frontal Cortex |
|  |  | 4.8 | Anterior Insula |
|  |  | 5.8 | Lateral Orbitofrontal Cortex |
| Cannula 3 | | | |
| 2.0 | 0.6 | 2.0 | A24b |
|  |  | 3.0 | A24a |
|  |  | 3.5 | A33 |
|  |  | 6.6 | Nucleus Accumbens Shell |
| Cannula 4 | | | |
| 2.0 | 1.8 | 1.5 | Motor Area 2 |
|  |  | 4.5 | Dorsomedial Striatum |
|  |  | 5.7 | Ventromedial Striatum |
|  |  | 6.9 | Nucleus Accumbens Core |
| Cannula 5 | | | |
| -2.5 | 0.7 | 1.3 | A30 |
|  |  | 2.3 | A29 |
|  |  | 4.7 | Mediodorsal Thalamus |
|  |  | 5.7 | Centro-median Thalamus |
| Cannula 6 | | | |
| -2.5 | 4.9 | 5.1 | Dorsolateral Striatum |
|  |  | 6.1 | Dorsolateral Striatum |
|  |  | 7.1 | Central Amygdala |
|  |  | 8.1 | Basolateral Amygdala |
| Cannula 7 | | | |
| -3.5 | 2.5 | 1.4 | Posterior Parietal Cortex |
|  |  | 2.5 | CA1 |
|  |  | 3.5 | CA3 |
|  |  | 8.0 | Subthalamic Nucleus |
| Cannula 8 | | | |
| -6.0 | 3.5 | 1.0 | Visual Cortex V1 |
|  |  | 1.7 | Visual Cortex V1 |
|  |  | 2.8 | Dorsal Subiculum |
|  |  | 3.7 | Dentate Gyrus |

**Supplemental Figure 1**. Histological Verification of LFP Target Sites. (A) Schematics of coronal brain sections with the location of 32 electrodes (8 cannula) marked. The coronal sections are modified from a rat brain atlas (Paxinos & Watson, 2013). Each cannula contains four wires each targeting a unique DV location. The identified centers of each electrode for all subjects are marked with pink dots when visible. An example thionin-stained coronal slice at the corresponding AP location is shown for each cannula with magnification of each track. (B) The table includes the AP, ML, DV coordinates based on bregma for all 32 electrodes and their corresponding nomenclature (Paxinos & Watson, 2013).

| Proportion of Large Reward Choice |  | **df** | ***F*** | ***p*** |  | **b** | ***t*** | ***p*** |
| --- | --- | --- | --- | --- | --- | --- | --- | --- |
|  | Subject | 13, 82 | 4.60 | **<.001** |  | 0.65 | 7.19 | **<.001** |
|  | Delay | 5, 82 | 18.81 | **<.001** |  | 0.56 | 8.22 | **<.001** |
|  | Subject*Delay | 52, 82 | 0.72 | 0.274 |  |  |  |  |

**Supplemental Table 1**. Statistical parameters from temporal discounting behavioral analysis.

| Fixed Effects |  | **df** | ***F*** | ***p*** |  | **b** | ***t*** | ***p*** |
| --- | --- | --- | --- | --- | --- | --- | --- | --- |
| **LOFC Power** | Trial | 1, 164.43 | 5.54 | **0.020** | Large v. Small | 0.12 | 1.72 | 0.088 |
|  | Frequency | 5, 52.82 | 2.90 | **0.022** |  |  |  |  |
|  | Trial*Frequency | 5, 164.42 | 1.28 | 0.274 |  |  |  |  |
| **Beta Power** | Trial | 1, 2555.14 | 2.13 | 0.145 |  |  |  |  |
|  | Delay | 5, 2544.84 | 22.52 | **<0.001** |  |  |  |  |
|  | Electrode | 11,2546.27 | 1.72 | 0.063 |  |  |  |  |
|  | Trial*Electrode | 11,2546.24 | 0.22 | 1.000 |  |  |  |  |
|  | Trial*Delay | 5,2550.09 | 27.60 | **<0.001** | Large v. Small | 0.44 | 2.30 | **0.022** |
|  | Electrode* Delay | 55, 2546.24 | 0.44 | 1.000 |  |  |  |  |
|  | Trial*Delay* Electrode | 55, 2546.23 | 0.24 | 1.000 |  |  |  |  |

| Random Effects |  | **Random Effect  Variance** | **Wald Z** | ***p*** |
| --- | --- | --- | --- | --- |
| **LOFC Power** | Frequency \| Subject | 3.30 | 3.09 | **0.002** |
|  | Time | 0.02 | 0.15 | 0.882 |
| **Beta Power** | Subject | 2.70 | 2.14 | **0.033** |
|  | Time | 1.01 | 0.98 | 0.326 |

**Supplemental Table 2**. Statistical parameters from multi-channel electrophysiology analyses. Linear mixed model fixed and random effects.

| Subject | High Reward Delay | Impedance | Current | Electrode |
| --- | --- | --- | --- | --- |
| R321 - *female* | 5s | 30kOhm | 80µA | ALM |
| R311 - *male* | 5s | 65kOhm | 40µA | A32D |
| R309 - *male* | 10s | 40kOhm | 40µA | A32V |
| R292 - *female* | 5s | 85kOhm | 35µA | Ains |
| R297 - *female* | 2s | 30kOhm | 80µA | vOFC |
| R271 - *male* | 5s | 30kOhm | 80µA | VMS |

**Supplemental Table 3**: Stimulation Parameters
